# Worker role perceptions and work participation among people with mental health issues taking part in interventions focusing on everyday life

**DOI:** 10.3233/WOR-220582

**Published:** 2023-10-19

**Authors:** Mona Eklund, Martin Bäckström

**Affiliations:** a Department of Health Sciences, The Mental Health, Activity and Participation (MAP) Group, Lund University, Lund, Sweden; b Department of Psychology, Lund University, Lund, Sweden

**Keywords:** Key words: Activities of daily living, mental illness, occupational therapy, outcomes, satisfaction

## Abstract

**Background:**

Knowing whether interventions addressing everyday life as a whole can affect work readiness for people with severe mental health issues would be important for how to develop support.

**Objective:**

To compare two groups of people with mental health problems, receiving either of two types of 16-week activity-based interventions, Balancing Everyday Life (BEL) or Care as Usual (CAU), regarding work readiness in terms of perceived worker role and satisfaction with recent work experience. Changes from baseline (T1) to completed intervention (T2) and a six-month follow-up (T3) and variables of potential importance to changes were also explored.

**Methods:**

This cluster RCT recruited participants for BEL (*n* = 133) and CAU (*n* = 93) from specialized and community-based psychiatry. Questionnaires addressing work readiness and potentially influencing variables (sociodemographic, clinical, type of intervention, work experience, non-work activity factors, social interaction and self-esteem) were used. Mixed model regression analyses were employed.

**Results:**

Positive changes occurred for both groups in one worker role aspect (resources for a future worker role) and in satisfaction with recent work participation. Satisfaction with non-work everyday activities, having valued activities, and self-esteem were important for change in the work readiness variables, whereas intervention type, age, sex or general activity level were not.

**Conclusion:**

Both interventions yielded equally positive work readiness outcomes. Support that emphasizes engagement in satisfying and valued everyday activities and boosts self-esteem would be a potential way to help people with mental health issues develop work readiness in terms of the worker role and satisfaction with work participation.

## 1 Introduction

Work, paid or unpaid, is an important part of people’s lives and unemployment may cause poorer mental health [1, 2]. Work can bring experiences that are unique compared to other everyday activities, such as feeling that one is a person who contributes to society, is part of a team that produces something together, and provides for oneself and family [3]. Persons with mental health issues desire work just as people in general. However, paid work on the open employment market may not be the universally prevailing option, being as there are aspects of work, and types of work, that are detrimental to well-being [4]. Positive connotations in terms of providing for oneself and being part of a working community dissolve if work is seen as rigid, demanding, and stressful [5]. Moreover, fluctuations in service users’ state of mental health require some flexibility in the job demands [4–6], something that the employer is not always able or willing to arrange. Persons with mental health issues who have experienced open-market employment as too demanding may thus prefer semi-market work or other ways of feeling productive. Laws [7] problematized traditional boundaries of work and found that mental health service users included working towards personal recovery as something legitimate to do during the day, in addition to a variety of market and semi-market activities. The current study proceeds from this wider definition of work.

Work may be seen as one of many activities that structure people’s everyday life [8]. If open-market is not feasible for a person with mental health issues, other work-related and recovery-oriented activities can bring meaningfulness similar to that associated with work [9]. Interesting and invigorating leisure activities, social activities, taking care of one’s home and keeping to a healthy lifestyle were some examples given in the latter study.

Several attempts have been made to develop methods for support as regards work for people with mental health issues. Supported employment (SE) sets its sight on open-market employment and has been successfully implemented in various Western societies [10, 11]. SE is a method for vocational training building on work placement from day one, with backing from a SE supporter. The focus in SE is “place, then train”, contrary to what is known as traditional vocational rehabilitation, where the focus is “train, then place”. SE has been found to be the by far more effective method of the two with respect to coming into work, at least one day [10–12]. Furthermore, interventions based on activities of everyday life in a broad sense and supporting a balanced lifestyle have proven to be effective for return to work among people with mild mental health issues [13–15]. Whether an intervention focused on activity in that broad sense can affect work readiness factors for people with severe mental health issues does not seem to have been investigated, however, which was the rationale for the current study, which is part of a randomized controlled trial (RCT) evaluating the effectiveness of the Balancing Everyday Life (BEL) intervention compared to Care as Usual (CAU), also activity-based [16]. The activity- and recovery-oriented BEL intervention addresses a number of everyday activities, such as social activities, work, other productive activities, and leisure, but foremost self-chosen activities. CAU consisted of occupational therapy in accordance with best practice. The RCT showed that the BEL group improved more than the CAU group regarding engagement in everyday activities and psychosocial functioning, and at a six-month follow-up the BEL group had improved more on quality of life as well [16]. Given the emphasis put on work in most societies, it seems warranted to investigate work readiness factors in relation to the BEL intervention and CAU, even if work is only one of the activity themes comprising these interventions. Work can be addressed as both actual doing and as an experiential readiness aspect, both of which were targeted in this study.

### 1.1 Aim

The aim of this study was to compare two groups of people with mental health problems, receiving either of two types of activity-based interventions, BEL or CAU, regarding experiential aspects of readiness for work, in terms of perceived worker role and satisfaction with recent work participation. Part of the aim was to describe change in each of the groups regarding perceived worker role and satisfaction with work participation from baseline (T1) to completed intervention (T2) and a follow-up six months after completed intervention (T3). Another part of the aim was to explore variables of potential importance to changes in worker role perceptions and work participation, in terms of sociodemographic and clinical factors at baseline, previous actual work experience, recent actual work experience, type of intervention received, and changes in non-work activity factors, social interaction and self-esteem during intervention and the follow-up period.

## 2 Methods

The cluster RCT on which the current study is based was approved by the Regional Ethical Vetting Board in Lund, Reg. No. 2012/70. All procedures were in accordance with Swedish legislation regulating research on humans [17] and with the Helsinki Declaration of 1975, as revised in 1983 and 2004. The study was registered with ClinicalTrial.gov. Reg. No. NCT02619318.

### 2.1 The interventions

The BEL intervention was inspired by previous research on lifestyle interventions [13, 18] and descriptive studies on everyday life among people with mental health issues [9, 19–21]. BEL is a group-based (5–8 participants) 16-week program consisting of 12 sessions, one session a week, and 2 biweekly booster sessions. The length of sessions is 1***1/2–2 hours and each session has a theme, such as *my sources for meaning and motivation, activity balance, healthy living, work-related activities,* and *leisure and relaxation*. The sessions are composed of brief educational sections, exercises and discussions and include self-analysis of wanted activities. Goals and strategies for testing activities are developed, based on the self-analysis. Home assignments, in terms of testing targeted activities, are completed between sessions. The outcome of the assignment is evaluated in the next group session, and depending on the result, goals can be renegotiated or new goals set. Informal peer support among the BEL participants is encouraged as well. The intent is that the participants will have gained an ability to reflect on their own situation after having completed the BEL program, and have found strategies for steering their everyday life towards a desired direction, so that they feel they have a balance between rest and work, seclusion and social activities, etc., and find satisfaction and meaning in everyday life. The program also inspires the participants to keep working with the BEL material after the group has ended, on their own or together with other group members, in order to sustain possible progress made. The BEL program is led by one or (preferably) two licensed occupational therapists who have taken part in 2–3 day’s training program and use a structured BEL manual [22]. An implementation study indicated high fidelity to the manual and that dose delivered was as intended [23]. Although the BEL program is not specifically oriented towards work, many of the resources and abilities required to uphold the role as a worker are focused upon in the program.

The CAU intervention was standard occupational therapy given in specialized psychiatric settings or activity-oriented support provided in day centers in community-based psychiatry, in all cases provided by a licensed occupational therapist. The CAU often included some form of group intervention, where daily living skills, social skills, creative activities or leisure were targeted, while some occupational therapists who worked in specialized psychiatry provided individual therapy only. The occupational therapists adhered to principles for “best practice”, which could vary depending on the participants’ mental health status and support needs. Just as in the BEL intervention, many of the resources and abilities required for a worker role were addressed in the CAU. Some settings also had a specific focus on work in their program.

Similarities between the interventions concerned that both were activity-based and led by licensed occupational therapists with vast experience from working in mental health care (1–25 years), both BEL and CAU therapists were part of a team that could provide a range of interventions, and most participants also received either or both psychotropic medication and some form of supportive therapy from other team members.

### 2.2 Recruitment of settings and participants

**Fig. 1:**
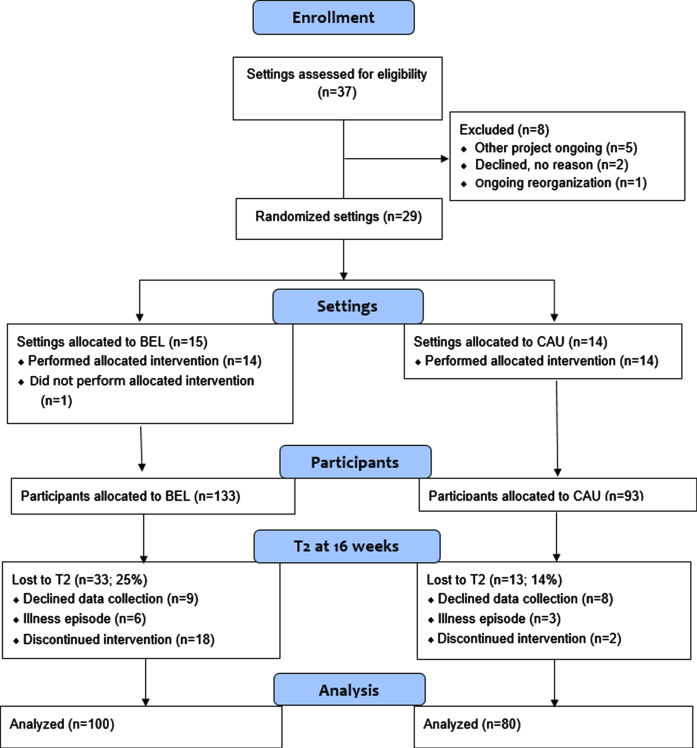
Chart showing inclusion and exclusion of settings and participants.

Using the means and standard deviations based on a previous study [24] applying the Satisfaction with Daily Occupations instrument (see below), and expecting a mean of 12.5 participants from each cluster and an ICC of 0.05 [25], we arrived at 65 participants in each group to detect a difference of 0.5 with 80% power at *p* < 0.05. We expected 25% attrition and aimed to include 95 participants from BEL settings and the same number from CAU settings. The different steps of including settings and participants are illustrated in Fig. 1. All outpatient units within general psychiatry, psychosis care, and community-based mental health centers in three Swedish regions were invited to the project, but eight were subsequently excluded because of ongoing projects or reorganization. Fifteen of the remaining 29 settings were randomized to the BEL arm and 14 to the CAU arm. One BEL setting then withdrew because of illness among staff.

Each consenting setting appointed an occupational therapist who would serve as a gatekeeper and identify eligible participants. Through information gained during team conferences and interviews with prospective participants, the gatekeeper helped to identify persons meeting the following criteria: perceiving imbalance among everyday activities (such as feeling over- or under-occupied or doing too little of valued activities), aged 18–65 years, substance use disorder not the main diagnosis, no comorbidity of dementia or developmental disorder, and sufficient knowledge of Swedish to complete the data collection. A broad range of psychiatric diagnoses were accepted, including psychoses, mood disorders and neuropsychiatric disorders. All eligible participants were invited and received oral and written information about the project from the gatekeeper. Those who chose to accept the invitation were subsequently contacted by a research assistant who made appointments for the data collection. The data collection took place in a quiet and private room in the respective settings and started with participants signing their informed consent. They received a small payment and had any travel expenses covered. A total of 226 participants entered the study; 133 from BEL settings and 93 from CAU settings.

A total of 226 participants were enrolled, 133 from BEL settings and 93 from CAU settings. A vast majority, about 90% in both groups, were born in Sweden. Further characteristics are presented in Table 1, showing that the participants were comparable on all known sociodemographic and clinical characteristics.

**Table 1:** Sociodemographic and clinical characteristics of the participants

Characteristics	BEL participants	CAU participants	*P*-value
	*N* = 133	*N* = 93
Gender (% women)	77	67	Ns.
Age (mean, SD)	40 (11)	40 (11)	Ns.
Living with partner (%)	30	26	Ns.
Has children living at home (%)	47	47	Ns.
Highest educational level (%)			Ns.
Nine-year compulsory school or lower	18	21
High school	59	60
College/university	23	19
Self-reported first diagnosis (%)			Ns.
Psychosis	19	24
Anxiety/bipolar/depressive disorders	52	50
ADHD/ADD	23	16
Other	6	10
Uses prescribed psychotropic medicine (%)	92	93	Ns.

Note: BEL = Balancing Everyday Life, CAU = Care as Usual.

From T1 to T2 there were 33 dropouts (25%) in the BEL group and 13 (14%) in the CAU group, which was a statistically significant difference (*p* = 0.047). Reasons for dropping out in the BEL group mostly concerned not completing the intervention, such as attending only the first group session. Dropping out of the study due to not wanting to complete the data collection or having an illness episode affected both groups similarly. Between T2 and T3, another 11 participants (8%) in the BEL group and 10 (11%) in the CAU group dropped out, which was not a statistically significant difference (*p* = 0.527).

### 2.3 Data collection

The research assistants who performed the data collection had substantial experience from working in mental health care and were trained as occupational therapists or psychologists. The data collection was performed in a secluded room in the setting and started with a background questionnaire developed specifically for the study. It consisted of questions about sociodemographic factors, work experience in the past, medication, self-reported diagnosis, and occurrence of perceived psychological and physical problems. Self-report questionnaires, repeated on all three measurement occasions, were used to address work factors, other everyday activities, social interaction, and self-esteem, as detailed below. All data collection was done on paper and participants filled out the questionnaires themselves. They could consult the research assistant if they found an item hard to understand, but the research assistants were careful not to influence the participants’ responses.

#### 2.3.1 Work factors

Two instruments were used to address work readiness factors. One of this addressed experiential and readiness aspects only, in terms of views on one’s worker role. The 16-item Worker Role Self-assessment (WRS) [26, 27] is a questionnaire based on self-reporting. It consists of statements that are rated according to five alternatives, ranging from 1 = “totally disagree” to 5 = “totally agree”. A higher rating indicates stronger beliefs/resources. WRS has two subscales (“beliefs in a future worker role” and “having worker role resources”), both of which have good construct and known-groups validity and satisfactory homogeneity [26]. The “beliefs in a future worker role” subscale includes items such as seeing the worker role as important, believing in a future working life for oneself, having goals for what to accomplish in working life, and getting support from family and health care services to gain a worker role. Items that form the “resources for having a worker role” subscale include, e.g., resilience, having good routines, awareness of skills and limitations, and taking responsibilities. WRS is based on the Model of Human Occupation, where the term role is defined as a set of behaviors, obligations and norms linked with a certain social situation [8], such as work.

The second measure to address work-oriented factors was the Satisfaction with Daily Occupations (SDO) instrument. It addresses four domains of everyday activities – work/studies, leisure, home management, and self-care [28, 29]. A version with 14 two-partite items was used. The first part concerns actual doing and asks whether the person currently performs the exemplified activity, response options being yes/no. Regardless of whether the answer to the first part of the item was yes or no, the second part asks about the person’s satisfaction with the activity, thus an experiential aspect. You can be satisfied or dissatisfied with something you currently do, but you can also be satisfied or dissatisfied with not being currently involved in an activity. The response scale for activity satisfaction has seven steps, from 1 = “worst possible satisfaction” to 7 = “best possible satisfaction”. The yes/no responses are compiled into an activity level score (range 0–14) and the satisfaction score into a satisfaction with everyday activities score (range 14 – 98). The SDO instrument has shown good construct validity, homogeneity and test-retest stability [28–30].

Two SDO items (being employed or admitted to an educational establishment at any point during the past two months, and currently involved in work or studies) were used to indicate recent actual work/study participation (possible score range 0 – 2) and satisfaction with actual recent work/study participation (possible score range 2 – 14). These two items were set to form subsets of the SDO items, satisfaction with recent work/study participation being included among the experiential work readiness factors and actual recent work experience as a possible influencing variable.

The remaining SDO items were compiled to form an estimate of everyday activities in general, as described below among the instruments used to reflect various variables of potential importance for change in the experiential work readiness variables.

#### 2.3.2 Non-work activity factors

Twelve of the SDO items formed an index reflecting general satisfaction with everyday activities. Two were from the work/study domain but did not address open-market employment. One concerned work/study-oriented rehabilitation and the other attendance at day centers. Three items addressed leisure (leisure on one’s own, organized leisure, cultural leisure), four involved management of one’s home (cooking & cleaning, gardening & repairs, planning the home chores, taking care of others) and three concerned self-care (personal hygiene, keeping fit, relaxation). The internal consistency reliability based on these twelve items and the current sample was α = .78.

A second general activity measure was the 18-item Occupational Value with pre-defined items (OVal-pd) [31]. It reflects the value a person perceives when engaged in everyday activities. Three value dimensions are included; concrete value, such as earning money or learning something new; socio-symbolic value, such as connecting with one’s culture or feeling strengthened in one’s identity; and self-reward value, which is when the activity in itself is the reward, bringing joy and satisfaction. The items take the form of statements and a four-point response scale is used, from 1 = “very seldom or never” to 4 = “very often”. OVal-pd has shown to be reliable and valid in different societal contexts [31–33].

#### 2.3.3 Social interaction

Social interaction was measured by one of the subscales from the self-report version of Interview Schedule for Social Integration (ISSI-SR) [34]. The subscale targeting availability of social interaction (AVSI) has been found to reflect a homogeneous factor with good construct validity [35]. The wording of items starts with “How many ...  ”, and is then followed by, e.g., “ ...   are you in contact with who have the same interests as you” and “ ...   friends do you have who come home to you anytime and feel at home“. The numbers given are subsequently coded into 1 or 0 for each item. Seven items were used for the current study: the six-item AVSI subscale, plus one item addressing availability of attachment. This means that the sum score could vary between 0 and 7, and internal consistency reliability based on the current study was *α*= .67.

#### 2.3.4 Self-esteem

The Rosenberg self-esteem scale was used to address self-esteem [36]. This ten-item scale measures a global sense of self-worth, and items cover different aspects of self-esteem, such as feeling like a valuable person and on a par with others. The present study used a yes/no response format proposed by Oliver and colleagues [37]. The Swedish version used in the current study has shown a one-component factor structure and good psychometric properties in terms of internal consistency, various aspects of construct and criterion validity, and sensitivity to change [38].

#### 2.3.5 Clinical factors

Self-reported diagnosis, type of intervention received, and level of functioning were the clinical factors included in the analyses. The participants’ self-reported diagnoses were subsequently categorized by a specialist psychiatrist according to ICD-10 [39]. This procedure has been found reliable in previous research [40]. The intervention types were BEL and CAU, as outlined above.

#### 2.3.6 Level of functioning

Global Assessment of Functioning (GAF) [41] was used to estimate level of functioning. A professional, in this study a research assistant, performs a rating on an interval from 0 to 100, where values of≥80 indicate good mental health and values≤50 indicate severe mental health issues. Two ratings are made, one for psychiatric symptom severity and one for psychosocial functioning. GAF has shown good concurrent validity and inter-rater reliability and is considered a valid summary of symptoms and social functioning [42]. The two ratings may be merged, but were analyzed separately in the current study.

### 2.4 Data analysis

Data formed three within-person levels, pertaining to baseline, completed intervention and the follow-up, but there were some missing data among levels. There were altogether about 15% missing values, and testing the data with the Little’s MCAR test revealed patterns that significantly departed from ‘missing completely at random’ (MCAR). We tested with independent Welch *t*-tests whether the participants with complete data differed significantly from participants with at least one missing value on at least one level. Only symptom severity revealed a difference, t(190) = 1.98, *p* = .05. Based on this result we performed mixed model analysis including all participants when they had contributed data from at least one level (mediation analysis demanded complete data sets).

We performed mixed model regression to investigate the change in work readiness (perceptions of the worker role and satisfaction with recent work participation) over the three levels: at baseline, at intervention completion and at follow-up. We calculated the intra-class coefficient 1 (ICC1) to give an estimate of the amount of between-person variance in the dependent variables, i.e., how much variance could be explained by differences between participants. Then we tested whether there was a significant change in the work readiness variables over the three within-person levels, and if change interacted with treatment type. Thereafter, we investigated whether the baseline estimates were related to change in the work readiness variables over the three levels, and also whether the baseline estimates interacted with change in work readiness. Lastly, we tested whether the baseline estimates could also be regarded as possible mediators of change in the work readiness variables over the three levels of baseline, completion and follow-up.

Standardized estimates will be reported to make interpretation easier for all analyses except the mediations. Belief in having a future worker role was somewhat skewed to the right, whereas resources for having a worker role was normally distributed. The distribution of satisfaction with recent work participation was more complicated; the distribution was almost rectangular, and participants tended to give the same response to both items that composed this variable, making some values more common. The skewness and kurtosis were however not exceptionally high.

## 3 Results

First, we investigated whether belief in having a future worker role and resources for having a worker role changed from baseline to follow-up in the study group as a whole. The ICC1 was .729 for belief in having a future worker role, .641 for resources for having a worker role, and .370 for satisfaction with recent work participation, suggesting that participants varied greatly on all dependent variables. It was found that belief in having a future worker role did not change, F(2,172.7) = .093, *p* > .05, *β*= .004 upon completion, or *β*= –.019 at follow-up. There was a significant change in resources for having a worker role, *F*(2, 176.3) = 17.184, *p* < .001. Compared to baseline, it was *β*= .157 higher (*p* < .005) after completed intervention, and at follow-up it was *β*= .37 higher (*p* < .001). Satisfaction with recent work participation also changed; from baseline to completed intervention it was *β*= .21 higher (*p* < .005) and to follow-up *β*= .31 higher (*p* < .005).

Table 2 shows descriptive statistics for the two worker role variables and satisfaction with recent work participation per intervention group. The only statistically significant difference between the groups concerned satisfaction with recent work participation at the follow-up, when the CAU group rated higher.

**Table 2:** Descriptive statistics for the two worker role variables and satisfaction with work participation in the BEL group and the CAU group; mean (SD)

	The BEL group	The CAU group	*P*-value
Belief in a future worker role
At baseline	26.5 (8.0)	25.5 (7.8)	Ns.
At completed intervention	26.2 (7.6)	26.2 (8.0)	Ns.
At follow-up	26.1 (7.9)	25.7 (9.4)	Ns.
Having resources for a worker role
At baseline	26 (5.1)	25.7 (5.0)	Ns.
At completed intervention	26.7 (4.7)	27 (5.3)	Ns.
At follow-up	27.6 (5.5)	28.2 (5.3)	Ns.
Satisfaction with recent work participation
At baseline	8 (3.7)	8.1 (4.0)	Ns.
At completed intervention	8.4 (3.9)	9.6 (3.6)	*p* < .05
At follow-up	9.4 (3.6)	9.1 (3.9)	Ns.

Note: BEL = Balancing Everyday Life, CAU = Care as Usual.

Next, we analyzed differences between the BEL group and the CAU group concerning change over time in worker role and satisfaction with recent work participation. There were no significant differences between the two treatment groups; both treatments seemed to have about the same effect on the two worker role variables, as well as on satisfaction with recent work participation. The coefficients from the interactions were; for belief in a future worker role: *β*= .02 and *β*= .03; for resources for having a worker role: *β*= .03 and *β*= .09; and for satisfaction with recent work participation: *β*= .27 and *β*= –.07 (first figure at completion, second at follow-up, all *p* > .05).

### 3.1 Baseline variables and belief in having a future worker role

All statistics are displayed in Table 3. We tested whether any of the baseline variables and the covariates explained the lack of significant change in belief in having a future worker role, but adding them to the model with only time did not change the result. However, a number of covariates were related to belief in having a future worker role in a cross-sectional perspective, namely age, recent work participation, symptom severity, psychosocial functioning, social interaction, and perceived activity value (all relations concern baseline data). We also tested interaction between time and the baseline variables. The only significant interactions found were for age in relation to belief in a future worker role at the follow-up, older participants rating relatively lower, and for having had a job in the past in relation to belief in a future worker role at follow-up, those having had a job rating somewhat higher. The scarcity of interactions suggests that the baseline variables did not work as covariates, and except for age and having had a job in the past, they did not moderate the ratings of belief in having a future worker role.

**Table 3:** Baseline main and interaction effects of belief in a future worker role; all effects are standardized

	Only main effect	Main effect with Int	Int at completion	Int at follow up
Age	–0.346***	–0.315***	–0.002	–0.123*
Sex	–0.036	–0.043	–0.016	0.042
Intervention type	–0.044	–0.062	0.022	0.043
Satisfaction w. non-work activities	–0.061	–0.057	–0.021	0.008
Activity level	0.011	0.022	–0.019	–0.021
Self-esteem	0.024	0.034	–0.052	0.017
Social interaction	0.148*	0.184**	–0.041	–0.091
Activity value	0.156**	0.192**	–0.040	–0.101
Symptom severity	0.132*	0.140*	–0.018	–0.012
Psychosocial functioning	0.157*	0.186**	–0.053	–0.053
Having ever had a job	–0.027	–0.063	–0.008	0.145*
Recent work participation	0.229***	0.251***	–0.032	–0.044
Depression	–0.106	–0.078	–0.070	–0.025

Note: Int = model with main and interaction. * = p < = .05, **< = .01 ***, *p* < .001.

### 3.2 Baseline variables and resources for having a worker role

Next, we tested whether baseline variables were related to resources for having a worker role in a cross-sectional perspective (statistics displayed in Table 4). The following variables were related to that worker role aspect: symptom severity, psychosocial functioning, satisfaction with non-work daily activities, activity level, self-esteem, social interaction, and perceived activity value. We also tested whether the baseline variables interacted with the amount of change in resources for having a worker role from baseline to completion and to follow-up. Satisfaction with non-work daily activities and perceived activity value interacted with change at completion, and the interaction suggested that higher baseline values were related to less positive change. Furthermore, age, a diagnosis of depression, and self-esteem interacted with change at follow-up; these interactions too suggested that higher values were related to less positive change. To summarize, in a cross-sectional perspective there were several variables with a rather strong relation to resources for having a worker role; i.e., the between-subject difference in that worker role variable could to some extent be explained by variables such as satisfaction with non-work daily activities, self-esteem and activity level. Not many of the baseline variables interacted with outcome in terms of resources for having a worker role (cf. the two right-most columns in Table 4), high levels on baseline variables being related to somewhat worse outcomes.

**Table 4:** Baseline main and interaction effects of resources for having a worker role; all effects are standardized

	Only main effect	Main effect with Int	Int at completion	Int at follow up
Age	0.096	0.117	0.041	–0.131*
Sex	–0.096	–0.124	0.067	0.028
Intervention type	0.008	–0.025	0.033	0.086
Satisfaction w. non-work activities	0.390***	0.440***	–0.137*	–0.033
Activity level	0.328***	0.362***	–0.050	–0.072
Self-esteem	0.381***	0.447***	–0.110	–0.126*
Social interaction	0.161**	0.183**	–0.024	–0.056
Activity value	0.345***	0.431***	–0.141*	–0.187**
Symptom severity	0.191***	0.192**	0.008	–0.015
Psychosocial functioning	0.228***	0.254***	–0.060	–0.036
Having ever had a job	–0.067	–0.076	0.005	0.028
Recent work participation	0.094	0.075	–0.018	0.071
Depression	0.083	0.115	0.006	–0.128*

Note: Int = model with main and interaction. * = p < = .05, **< = .01 ***, *p* < .001.

### 3.3 Baseline variables and satisfaction with recent work participation

Next, we tested whether baseline variables were related to satisfaction with recent work participation in a cross-sectional perspective (statistics displayed in Table 5). The following variables were related to satisfaction with recent work participation at both completion and follow-up: symptom severity, psychosocial functioning, satisfaction with non-work daily activities, age, self-esteem, social interaction, and perceived activity value. We also tested whether the baseline variables interacted with the amount of change in satisfaction with recent work participation from baseline to completion and to follow-up, and findings showed that satisfaction with non-work daily activities, activity value, and recent actual work participation interacted with change at completion. For all baseline variables, higher values were related to less positive change. In addition, age and satisfaction with non-work daily activities interacted with change at follow-up, again suggesting higher values to be related to less positive change. To summarize, in a cross-sectional perspective there were several variables with a rather strong relation to satisfaction with recent work participation, and the relations concerned baseline covariates that were similar to those found for resources for having a worker role.

**Table 5:** Baseline main and interaction effects of satisfaction with recent work participation; all effects are standardized

	Only main effect	Main effect with Int	Int at completion	Int at follow up
Age	0.176***	0.251***	–0.042	–0.215**
Sex	0.060	0.069	–0.024	–0.006
Intervention type	0.044	0.015	0.133	–0.037
Satisfaction w. non-work activities	0.265***	0.404***	–0.243**	–0.221**
Activity level	0.010	0.035	–0.042	–0.045
Self-esteem	0.218***	0.283***	–0.122	–0.098
Social interaction	0.048	0.015	–0.011	0.130
Activity value	0.165**	0.254***	–0.205*	–0.107
Symptom severity	0.151**	0.179**	–0.043	–0.050
Psychosocial functioning	0.212***	0.227***	–0.038	–0.012
Having ever had a job	–0.022	–0.050	0.067	0.030
Recent work participation	0.170**	0.247***	–0.159*	–0.097
Depression	0.080	0.138*	–0.141	–0.054

Note: Int = model with main and interaction. * = p < = .05, **< = .01 ***, *p* < .001.

### 3.4 Mediation of change in resources for having a worker role and satisfaction with recent work participation

Several of the covariates were also estimated at completion and follow-up. These may be used as mediators of the identified change in resources for having a worker role and satisfaction with recent work participation. To mediate the treatment effect, change in the covariates must correlate with change in worker role and satisfaction with recent work participation, respectively. Table 6 displays the statistics for all included mediators to resources for having a worker role and satisfaction with recent work participation. For both of the dependent variables, satisfaction with non-work daily activities, self-esteem and perceived activity value were the strongest mediators of the treatment effect at both completion and follow-up. In addition, social interaction, psychosocial functioning and symptom severity mediated the change, but at a somewhat lower strength. Based on the information in Table 6 the following was shown: generally, columns A1 and A2 show that the mediators all increased from baseline to completion and follow-up, and these increments were strongest for the follow-up. In addition, there were rather strong relations between the mediators and the two dependent variables (columns B1 and B2 in Table 6). The significant indirect effects are shown in columns Ind1 (at completion) and Ind2 (at follow-up). The reduction in the estimated increase from baseline to completion of treatment and follow-up (columns Prop1 and Prop2 in Table 6) revealed only a partial mediation of the treatment effects. To summarize, the analyses especially support satisfaction with non-work daily activities, self-esteem and perceived occupational value as possible mediators to the treatment effects in terms of resources for having a worker role and satisfaction with recent work participation.

**Table 6:** Possible mediators to change in resources for having a worker role and satisfaction with recent work participation. All coefficients are based on unstandardized variables

	A1	A2	B1	B2	Ind1	Ind2	Prop1	Prop2
*Resources for having a worker role*								
Satisfaction w. non-work activities	3.74	4.88	0.15	0.15	0.54*	0.75***	0.77	0.38
Activity level	0.38	0.31	0.51	0.68	0.20*	0.21*	0.25	0.10
Self-esteem	0.11	0.20	3.52	3.58	0.38*	0.72***	0.47	0.38
Social interaction	0.16	0.42	0.51	0.56	0.08	0.24***	0.10	0.12
Activity value	2.28	3.46	0.22	0.21	0.50*	0.71***	0.72	0.36
Symptom severity	1.63	2.99	0.07	0.09	0.12*	0.27***	0.15	0.14
Psychosocial func.	3.26	5.82	0.05	0.08	0.16*	0.44***	0.21	0.22
*Satisfaction with recent work experience*								
Satisfaction w. non-work activities	3.87	4.36	0.10	0.12	0.39***	0.52***	0.48	0.44
Activity level	0.40	0.33	0.06	0.07	–0.02	0.02	0.02	0.02
Self-esteem	0.12	0.20	1.50	1.72	0.18**	0.34***	0.22	0.30
Social interaction	0.16	0.43	0.13	0.26	0.02	0.11*	0.02	0.09
Activity value	2.55	3.21	0.07	0.09	0.19**	0.28***	0.22	0.23
Symptom severity	1.65	2.97	0.04	0.05	0.06	0.15***	0.06	0.13
Psychosocial func.	3.14	5.77	0.04	0.05	0.13*	0.31***	0.15	0.27

Note: 1 = completed treatment, 2 = follow-up, *A* = relation to mediator, *B* = mediator to dependent, Ind = indirect effect, Prop = proportion of variance mediated, * = p < = .05, **< = .01 ***, *p* < .001.

A model was tested including all possible mediators, with the intent to investigate whether the mediation would be stronger. This would suggest that each of the mediators could explain unique variance of the treatment effect in resources for having a worker role. The model revealed four significant mediators, namely satisfaction with non-work daily activities, activity level, self-esteem, and perceived activity value. The coefficients for the treatment effect at completion was *B* = –0.05 (*p* > .05) and at follow-up *B* = 0.63 (*p* = .052); in other words, when using all mediators the reduction in the treatment effect approached zero, suggesting almost total possible mediation. Approximately the same reduction (no significant difference between the models, *p* > .05) was achieved with only satisfaction with non-work daily activities (*B* = 0.09, *t* = 5.65, *p* < .001), self-esteem (*B* = 2.32, *t* = 6.51, *p* < .001), and perceived activity value (*B* = 0.13, *t* = 4.24, *p* < .001) included in the model.

Analysis of mediation of satisfaction with recent work experience with all possible mediators resulted in total mediation, e.g. the treatment effect was not significant, *B* = 0.31, *p* > .05 and *B* = 0.35, *p* > .05 at completion and follow-up, respectively. The significant mediators were satisfaction with non-work daily activities (*B* = 0.13, *t* = 9.02, *p* < .001), activity level (*B*=–0.42, *t* = –5.01, *p* < .001), and self-esteem (*B* = 0.73, *t* = 2.42, *p* = .016). Note that the relation to activity level was negative, and since this variable did not have a significant univariate relation to satisfaction with recent work experience the result suggests a suppressed relation. A model with only satisfaction with non-work daily activities as a correlate also mediated the changes in satisfaction with recent work experience, pointing to non-significant treatment effects at both completion and follow-up.

Since the results for resources for having a future worker role and satisfaction with recent work experience were very similar, we investigated the relationship between the two. The correlations at the three measurement points were *r* = 0.09, *r* = 0.07, and *r* = 0.04, respectively, all *p* > .05.

## 4 Discussion

The BEL intervention and CAU showed similar outcomes in terms of the two worker role aspects addressed in this study. Both groups changed their ratings in a positive direction regarding resources for having a worker role, but their beliefs in having a future worker role did not change, neither at completed intervention nor at the six-month follow-up. This is similar to previous research based on worker role outcomes, pointing to an item addressing abilities of relevance for having a worker role as sensitive to change. That aspect, but no other worker role aspect, was indicative of return to work [43]. The current findings also resonate well with the character of the two interventions. None of these addressed work in particular, but the activity-focus that both interventions had in common, as well as the group format with exercises and discussions, entailed training in social and practical skills of relevance for working life. It may thus be seen as logical that resources for having a worker role were influenced by the intervention, but belief in actually having a worker role in the future was not. The participants in the current study rated their worker role similarly to other groups with mental illness (young people with psychosis/ people with substance use disorder /newly arrived immigrants) [44].

The result pattern obtained in this study makes it interesting to examine if factors other than the interventions influenced and interacted with perceived worker role. Regarding beliefs in a worker role, the lack of change could not to any great extent be explained by interactions with correlates; only younger age and having previously had experience of work were related to a more positive development in this worker role aspect. These findings largely concur with research addressing work rehabilitation, which is more extensively studied than perceptions of the worker role. For example, having previously had a job and younger age seem to be important factors for positive outcomes after vocational training for people with severe mental illness [45, 46].

Relations and mediation between the covariates and the two outcomes where a change was identified – resources for having a worker role and satisfaction with recent work participation – were very similar. One might suspect a substantial correlation between these two outcomes; in fact, however, the association was close to zero. The possible mediators identified as important for both outcomes were, in particular, change in satisfaction with non-work daily activities, self-esteem, and perceived activity value, and for all of these, increased ratings over the measurement points were related to better outcomes on the work readiness variables. Altogether, change in your satisfaction with everyday life and how you value what you do seemed more important than change in symptomatology.

Focusing on resources for having a worker role, the current findings indicated interaction effects for some of the variables. Higher ratings on satisfaction with non-work everyday activities, activity value and self-esteem, and a diagnosis of depression were related to decreased ratings on resources for having a worker role. The negative relationships with activity and self-esteem are somewhat surprising findings; positive relations between satisfaction with non-work everyday activities and positive outcomes among people with mental health issues have otherwise been found in previous work addressing personal recovery [47] and well-being [48]. A possible explanation could be that participants who were originally worse off in these respects benefitted more from the interventions. Depression as a negative factor for work outcomes is on the other hand more expected and a prominent finding in research [49], which stimulated Johanson and colleagues to develop an adapted version of supported employment (SE) for people with depression [50], otherwise used with people with a variety of severe mental illnesses [51].

### 4.1 Implications for support

Interventions like those targeted in the current study seem valuable to increase work readiness in terms of satisfaction and general resources for entering or maintaining a worker role, despite that participants were seemingly unprivileged in relation to positive work predictors. Hansen and colleagues found that among the most important positive factors for returning to work after illness were that the individual held expectations of having work in the future and that the illness was some kind of somatic disorder [52]. The participants in the current study showed the opposite; the most common diagnosis was a mood disorder. People with depression have been found to be at risk of poor vocational outcomes compared to people with a somatic disorder [49], and the current study showed that they were also disadvantaged compared to people with other diagnoses, such as psychosis and neuropsychiatric disorders. This needs to be acknowledged in work-oriented support, and supported employment specifically adapted for people with affective disorders, as developed by Bejerholm and colleagues [12], would be a suitable method. Whereas supported employment addresses work per se, the current study focuses on a type of work readiness. Work readiness in terms of perceptions of one’s worker role has been seen as an indicator of return to work potential in the supported employment context [53]. Furthermore, one aspect of the worker role, “expectation of job success”, has shown to be predictive of return to work after sick leave [27, 43]. This is consistent with a recent review concluding that positive return to work expectations are the best predictor of return to work [54]. Thus, a focus on work readiness, in terms of for example people’s view of their worker role, would be an important component in all types of work rehabilitation.

The fact that improvements on non-work daily activities in terms of satisfaction and activity level, together with self-esteem, mediated most of the increments in resources for having a worker role and satisfaction with recent work participation opens up for additional prospects in relation to work-related support. Research has revealed a reciprocal influence between self-esteem and various experiences related to work (such as job satisfaction and job stressors), where the influence of self-esteem on future work experiences was somewhat larger than the importance of work experiences for future self-esteem [55]. In line with this, it has been suggested that developing self-esteem should be considered in work rehabilitation [56], and the findings regarding mediators in the current study support that idea. Research on effects of non-work everyday activity on work readiness aspects seems scarcer, but the current findings on mediations suggest that addressing activity level in general is not enough; satisfying and valued daily activities are more important and have potential to boost resources for having a worker role and satisfaction with work experiences.

### 4.2 Methodological discussion

The cluster RCT design did not allow for blinding, but allocation concealment and providing identical research information to prospective participants in both groups were other measures to strengthen the methodology. Interviewer effect is a potential bias in non-blinded studies, but was a minor threat since the outcome measures were based on self-reports. This study used instruments that have been found reliable and valid in several studies. Two of them were used somewhat differently compared to the original instruments and their internal consistency was therefore tested on the current sample. Regarding SDO, 12 items were discerned to reflect non-work activities, and the alpha value was in the realm for satisfactory. However, the seven items from ISSI-SR used to assess social integration did not yield the requested lower limit of 0.7 [57], but was very close at 0.67.

The term ‘effect’ is used in a statistical sense in this study. Although this was a longitudinal study, the direction of relationships could not be ascertained. We postulated the work readiness variables as outcomes and a selection of actual circumstances and perceptions as potential mediators. It is possible, however, that the work readiness variables in fact influenced the variables set as mediators, and a mutual influence is likely. Nevertheless, it seems warranted to utilize the potential inherent in supporting towards satisfying and valued non-work activities and improved self-esteem, not least since there are often greater opportunities to provide such support in mental health care, compared to actual work experiences. When reasoning around possible avenues for support, generalizability is an important issue. The current study sample represents a diagnostically heterogeneous group of mental health care users, but the common denominator was that all attended specialized mental health outpatient settings and all needed support to manage everyday life. A related study, focusing only on the BEL intervention [58], concluded that the intervention was suitable for mental health care users with a variety of sociodemographic, clinical, and self-related attributes. The current findings corroborated that, and the results would be transferable to similar settings and users.

## 5 Conclusion

Despite that none of the interventions was specifically targeted towards work, participants in both intervention groups increased their satisfaction in general, including at the follow-up, concerning both work readiness and non-work factors. Non-work factors mediated the variance in resources for having a worker role and satisfaction with recent work participation, suggesting that support targeted at increasing participants’ satisfaction with non-work activities, the value they derive from activities, and their self-esteem could reinforce the type of work readiness outcomes addressed in the current study. Future research should address whether support for engagement in non-work activities and increased self-esteem could also facilitate actual work.

## Ethical approval

The study was approved by the Regional Ethical Vetting Board in Lund (Reg. No. 2012/70).

## Informed consent

Each participant gave his or her oral and written informed consent. Participants were informed that, without explanation, they could withdraw their consent at any time.

## Conflict of interest

The authors report no conflict of interests. The authors alone are responsible for the content and writing of the paper.
